# Coagulation profiles and platelet parameters among preeclampsia, eclampsia, and normotensive pregnant women attending Comprehensive Specialized Hospital maternity wards, Northwest Ethiopia

**DOI:** 10.1371/journal.pone.0328578

**Published:** 2025-07-21

**Authors:** Nigusie Alemu, Bisrat Birke Teketelew, Sintayehu Admas, Lyusira Marelgn, Yalweayker Eyayu, Berhanu Woldu

**Affiliations:** 1 Department of Hematology and Immunohematology, School of Biomedical and Laboratory Science, College of Medicine and Health Sciences, University of Gondar, Gondar, Ethiopia; 2 Department of Medical Laboratory Science, College of Medicine and Health Sciences, Mizan Tepi University, Mizan Tepi, Ethiopia; African Union Commission, ETHIOPIA

## Abstract

**Background:**

Hypertensive disorders of pregnancy affect about 10% of all pregnant women worldwide. A variety of hematological abnormalities may occur in women with pregnancy-induced hypertension. Thus, we aimed to compare coagulation profiles and platelet parameters among preeclampsia, eclampsia, and normotensive pregnant women in Northwest Ethiopia.

**Method:**

A comparative cross-sectional study was conducted at University of Gondar and Felegehiwot Comprehensive Specialized Hospital, maternity wards from June 20 to November 15, 2024. A total of 180 participants were recruited using the convenience sampling technique. Socio-demographic and clinical data were collected using a structured questionnaire and participants’ medical records. Eight milliliters of blood specimens were collected from each study participant. Coagulation profiles and platelet parameters were measured using a Genrui coagulation analyzer and an automated Mindray BC-5150 hematological analyzer, respectively. Data were entered into epi-data version 4.6.0.6 and exported to the SPSS version 26 software for analysis. Descriptive data were presented using tables and frequencies. One-way ANOVA and Kruskal-Wallis tests were used for normally distributed and skewed data, respectively. A p < 0.05 was considered statistically significant.

**Results:**

A total of 180 pregnant women were enrolled/included in this study. The median PT and aPTT showed prolongation in preeclampsia and eclampsia patients, as compared to normotensive pregnant women, indicating a significant difference (p < 0.001) and (P = 0.002) respectively. The mean ± SD of mean platelet volume and PDW showed higher values in preeclampsia and eclampsia patients, as compared to normotensive pregnant women, demonstrating both a significant difference (P < 0.001). On the other hand, the mean platelet count in preeclampsia and eclampsia patients, were lower than in normotensive pregnant women, with a significant difference (p < 0.001).

**Conclusion and recommendations:**

In this study, alteration of coagulation and platelet parameters were observed in preeclampsia and eclampsia pregnant womens compared to normotensive controls. Thus, a routine laboratory test of coagulation and platelet parameters for pregnant women with preeclampsia and eclampsia could be applicable to reduce the risk of coaglation and platelet abnormalities.

## Introduction

Pregnancy is a natural phenomenon in women that can come up with certain risks to the health of both the mother and baby [1]. Hypertension is among the common serious problems seen in 7–10% of pregnancies leading to maternal and perinatal mortality and morbidity [2]. Pregnancy-induced hypertension (PIH) is a common complication of pregnancy and its diagnostic criterion of blood pressure is at least 140/90 mmHg on two separate occasions ≥4 hours apart after the 20^th^ week of gestation [3]. The PIH is classified into gestational hypertension (without proteinuria), pre-eclampsia (with proteinuria), eclampsia (pre-eclampsia with convulsions), and chronic hypertension with superimposed preeclampsia [4].

The pathophysiology of PIH involves diffuse vasospasm caused by loss of the normal pregnancy-related refractoriness to vasoactive substances such as angiotensin and relative or absolute changes in the prostaglandin substances [5]. Increases of the vasoconstrictor thromboxane and decreases of the potent vasodilator prostacyclin characterize it, and vascular contraction increases systemic vascular resistance, increasing blood pressure. This vasospasm leads to intravascular volume constriction and reduced perfusion of most organs [6].

An abnormal trophoblastic invasion leading to abnormal implantation causes endothelial dysfunction, mediated by immunological and inflammatory factors [7]. These events result in a hemostatic imbalance, reflected mainly by excessive hypercoagulability, proteinuria, and other clinical manifestations [8]. The endothelial activation and dysfunction in preeclampsia and eclampsia are characterized by platelet hyperactivation and hypercoagulation, with increased tissue factor expression and activity and fibrinolysis deregulation [8,9].

Preeclampsia is a pregnancy-associated disorder with new-onset hypertension, which occurs most often after 20 weeks of gestation [10]. It is a multisystem pregnancy disorder characterized by variable degrees of placental malperfusion, with the release of soluble factors into the circulation [11]. Organs affected by pre-eclampsia include the brain, eyes, liver, kidneys, and the hematological system (causing hemolysis, thrombocytopaenia, or coagulopathy) [6,10,12].

Clinical risk factors for pre-eclampsia are chronic hypertension, pregestational diabetes, family history of pre-eclampsia, previous placental abruption, and nulliparity [13]. These factors cause maternal vascular endothelial injury, which results in hypertension, multi-organ injury, and placental disease can cause fetal growth retardation and stillbirth [14]. However, the pathogenesis of pre-eclampsia is only partially understood and it is related to disturbances in placentation at the beginning of pregnancy, followed by generalized inflammation and progressive endothelial damage [15].

Eclampsia is a severe complication of preeclampsia, which is characterized by the occurrence of generalized seizures in the pregnancy or in the postpartum period [15]. Like that of pre-eclampsia, the pathogenesis of eclampsia remains largely unknown, and 5%–8% of women with pre-eclampsia can progress to eclampsia in developing countries. Hemolysis, elevated liver enzyme, and low platelet count syndrome occur in 10%–20% of women with eclampsia and are associated with substantial, widespread endothelial damage [16].

Alterations in the hemostatic system, including endothelial cell damage, platelet activation, and enhanced intravascular thrombin generation, are the major pathophysiologic events in pre-eclampsia and eclampsia [17]. Endothelial dysfunction will lead to altered levels of fibrinogen, activated partial thromboplastin time (aPTT), prothrombin time (PT), fibrin degradation products, and D-Dimers [18,19]. Alterations of endothelial cells and fibrin deposition in microvasculature lead to enhanced activation of the coagulation cascade and impaired fibrinolysis associated with multiple organ dysfunctions [20]. Among the hematological changes that occur in preeclampsia and eclampsia, thrombocytopenia is the most widely recognized, and its degree increases with advancing the disease severity [21].

Prothrombin time and aPTT are the most commonly performed tests in coagulation profiles to detect coagulation defects for extrinsic and intrinsic coagulation cascade pathways respectively. Both PT and aPTT are considered functional tests as they measure enzymatic activities that lead to clot formation [22]. Previous studies have shown that platelet test parameters play an important role in the pathogenesis of preeclampsia and eclampsia. Platelet indices involving the plateletecrit, Mean Platelet Volume (MPV), Platelet Distribution Width (PDW), platelet large cell ratio, and platelet concentration are a group of derived platelet parameters obtained from the automated complete blood count [23].

Several coagulation and platelet parameters are used to measure the derangement of the hemostatic system in both preeclampsia and eclampsia, like, PT, aPTT, the platelet count, MPV, the platelet count to MPV ratio, and PDW [24]. Therefore, we aimed to compare the coagulation profiles and platelet parameters among preeclampsia, eclampsia, and normotensive pregnant women at the University of Gondar Comprehensive Specialized

Hospital and Felege-hiwot Comprehensive Specialized Hospital maternity wards, Northwest Ethiopia.

## Methods and materials

### Study design, setting, and period

A comparative cross-sectional study was conducted from June 20 to November 15, 2024, at the University of Gondar Comprehensive Specialized Hospital (UoG-CSH) and Felege-Hiwot Comprehensive Specialized Hospital (FHCSH) antenatal care (ANC) units and maternity wards, Northwest Ethiopia. The study was conducted at the ANC units and maternity wards of UoG-CSH and FHCSH, Northwest Ethiopia. The UoG-CSH is a teaching Hospital located in Gondar town, 738 km from Addis Ababa, Ethiopia. It serves more than 7 million people in Gondar town and surrounding areas. The hospital has a larger ANC unit, and the delivery ward of the hospital has 4 rooms and 54 beds [25]. The FHCSH is located in Bahr Dar town, the capital of the Amhara region. It is located 565 km away from Addis Ababa, the Capital city of Ethiopia. The hospital is giving services to more than 5–7 million people per year in the western part of the region as a referral Hospital. The hospital’s capacity has more than 350 beds of which 55 are allocated for the Department of Obstetrics and Gynecology, about 3,521 mothers attend antenatal care each year [26].

### Source Population

All pregnant women diagnosed with preeclampsia and eclampsia at the UoG-CSH and FHCSH maternity wards.

### Study Population

Pregnant women diagnosed with pre-eclampsia and eclampsia and ≥20 weeks of gestation at UoG-CSH and FHCSH maternity wards. On the other hand, the normotensive pregnant women visiting ANC units during the study period were considered as control group.

### Inclusion and Exclusion Criteria

Pregnant women confirmed with pre-eclampsia and eclampsia at ≥20 weeks of gestation were included in the study as cases. Normotensive pregnant women visiting ANC units and matched for age and gestational age weeks with cases were included as controls. Preeclampsia and eclampsia patients with co-existing conditions: systemic diseases, like the history of hypertension, diabetes mellitus, history of thrombosis, hepatic disease, heart disease, history of renal disease, hepatitis B or C viruses, Human Immuno-deficiency Virus (HIV), malaria, and intestinal parasite infection, any hematological malignancies, cases taking medications, like anticoagulant drugs (warfarin, heparin, and aspirin) within two weeks were excluded. Normotensive pregnant women who were positive for malaria, intestinal parasites, HIV, hepatitis B or C viruses were excluded from the study. Moreover, normotensive pregnant women who have a history of hypertension, a family history of bleeding, severe bleeding within three months, diabetes mellitus, heart disease, history of renal disease, hepatic disease, malaria, any hematological malignancies, or controls taking medications, like anticoagulant drugs within two weeks were excluded.

### Sample Size determination and Sampling technique

According to rules of thumb that have been recommended by van Voorhis and Morgan, 30 participants per group are required to detect real differences, which can lead to about 80% power in a comparative study [27]. To enhance the study’s ability to detect the real difference, the required sample size of 30 individuals has been doubled, resulting in the inclusion of 60 individuals in each of the three study groups. A total of 60 pre-eclampsia patients, 60 eclampsia patients, and 60 normotensive individuals were recruited (using convenience, i.e., sequential sampling technique) from the UoG-CSH and FHCSH ANC units and maternity wards.

## Measurement of variables

**Normotensive pregnant women:** Is a pregnant women whose SBP between 90 and 120 mmHg and DBP between 60 and 80 mmHg [28].

**Pre-eclampsia:** is defined as SBP ≥ 140 and/or DBP ≥ 90 mmHg on two measurements together with proteinuria of ≥1+ by dipstick in random urine on two occasions at least 4 hours apart sample after 20 weeks of pregnancy [29].

**Eclampsia:** is defined by new-onset SBP ≥ 140 and/or DBP ≥ 90 mmHg with convulsion/seizures on two measurements together with proteinuria of ≥1+ by dipstick in random urine on two occasions at least 4 hours apart sample after 20 weeks of pregnancy [29].

**Parity**: is defined as the number of deliveries with a gestational age of 24 weeks or more (at ≥6 months gestation), regardless of whether the child was born alive or stillborn [30].

**Gravidity**: is defined as the number of times that a woman has been pregnant, including all live births and pregnancies that terminated at <6 months or lost due to stillbirths or miscarriages [31].

**Thrombocytosis and Thrombocytopenia** are considered when the blood platelet count is > 300 × 10^3^/µL and < 100 × 10^3^/µL, respectively (Based on Mindray BC-5150 reference range).

**Abnormally high and low MPV:** are considered when MPV > 11 fL and < 7 fL, respectively (Based on Mindray BC-5150 reference range).

**Abnormally high and low PDW:** are considered when PDW > 17 fL and < 9 fL, respectively (Based on Mindray BC-5150 reference range).

**Prothrombin Time (PT):** was measured to assess the integrity of the extrinsic system and factors common to both systems, with a normal range of 13–17 seconds (depending on the Generui CA-51 coagulation analyzer) [32].

**Activated Partial Thromboplastin Time (aPTT):** was measured to evaluate the integrity of the intrinsic system and the common components, with a normal range of 23–45 seconds. (Depends on the Generui CA-51 coagulation analyzer) [32].

**Prolonged and short PT:** are defined as PT > 17 seconds and < 13 seconds respectively (Depends on the Generui CA-51 coagulation analyzer).

**Prolonged and short aPTT:** are defined as PT > 17 seconds and < 13 seconds respectively (Depends on the Generui CA-51 coagulation analyzer).

### Socio-demographic and clinical data collection

Socio-demographic characteristics of the study participants were collected using a pre-tested structured questionnaire via a face-to-face interview approach by trained clinical midwives (S1 Table). Clinical and other relevant data were obtained from the participants’ medical records and charts using the data extraction sheet. Blood pressure was measured from the left arm at the heart level using a mercury sphygmomanometer and stethoscope in the sitting position after at least 15 minutes of rest. Anthropometric data were collected following the WHO stepwise approach to the preeclampsia and eclampsia surveillance manual and by trained clinical midwives. Body weight (Kg) to the nearest 0.1 kg and height (m) to the nearest 0.1 cm were measured, without shoes and in light clothing, using a portable weight scale, which has an attached height scale. The BMI was calculated as body weight in kilograms (Kg) divided by the square of height in meters (m^2^). Mid-upper arm circumference was measured on a straight arm midway between the tip of the shoulder and the tip of the elbow.

## Laboratory methods

### Blood specimen collection

A total of 8 mL of venous blood was collected from each participant via syringe and needle, then aliquoted into three tubes: 3 mL in a K₂-EDTA tube for platelet (PLT) analysis, 3 mL in a 3.2% trisodium citrate tube for PT and aPTT coagulation testing, and 2 mL in a serum separator tube (SST) for HIV, HBV, and HCV serological screening.

## Laboratory analysis

### Platelet parameters analysis

Platelet parameters, including PLT count, MPV, and PDW, were performed by laboratory technologists and analyzed using an automated hematological analyzer (Mindray BC-5150, *Shenzhen*, *China*). Platelet counting analysis was done using the electrical impedance principle involves passing a blood sample through a small aperture with electrodes on either side. As platelets pass through this aperture, they disrupt the electrical current, and the resulting voltage pulse is measured. The size and number of these pulses are used to determine the platelet count. The standard operating procedures, daily maintenance, weekly maintenance, and internal quality control procedures for the analyzer were strictly adhered to throughout the research process.

### Coagulation parameter analysis

Basic blood coagulation profile tests (PT and aPTT) were performed by laboratory technologists using the semi-automated Genuri coagulation analyzer *(CA51, China).* This analyzer uses the optical detection (turbido-metric) principle at a temperature of 37°C. This method relies on measuring the change in light transmission through a sample as a clot forms, which is a key indicator in coagulation testing. During the entire research process, strict adherence to the standard operating procedures and internal quality control procedures of the Genrui coagulation analyzer was consistently followed.

### Data management and quality control

All the relevant sociodemographic and clinical data were collected by a well-trained clinical midwives, while blood samples were collected by an experienced laboratory technologist. Sample collection, processing, and laboratory testing were performed in accordance with the prepared standard operating procedures. Quality control tests were implemented following standard operating procedures. Adherence to specified conditions, such as temperature, time, and guidelines for sample-reagent mixing, was striclly followed. The quality of platelet parameter analysis were strictly followed during analysis with the incorporation of internal quality controls encompassed of high, medium, and low-level standards. Analyzer background checks were performed before sample analysis as part of the test procedure. (pre-tes, supervision)

### Data analysis and interpretation

Data was entered to epi-data version 4.6.0.6 and exported to IBM SPSS version 26 software for analysis. The Kolmogorov-Smirnov normality test was run to check the distribution of coagulation profiles and platelet parameters and the Levene’s statistic test was used to test the homogeneity of variances. Descriptive analysis were expressed using frequency, percentage, median, interquartile range (IQR), mean and standard deviation (SD). Platelet parameters were compared between case and control groups using a one-way ANOVA test followed by a Tukey multiple comparison test. Whereas, coagulation profiles were compared between case and control groups using the Kruskal-Wallis H test. The level of statistical significance difference was set at a 95% confidence interval with a p-value of less than 0.05.

### Ethical statements

The study protocols were reviewed and approved by the School of Biomedical and Laboratory Sciences’ ethical review committee with reference number (SBMLS/767/2024). A permission letter was obtained from the UoG-CSH and FHCSH Chief Clinical Directors. A written informed consent was obtained from each study participant, and a unique identification code was used to ensure the confidentiality of the study participants. Abnormal findings such as severe thrombocytopenia and abnormal prolongation of PT and aPTT among pre-eclampsia and eclampsia pregnant womens were communicated to their physicians for appropriate management and treatment services.

## Results

### Sociodemographic characteristics of study participants

A total of 180 study participants were included in this study (120 cases and 60 controls). Among the enrolled study participants 121 (67.2%) were from urban, and 32 (17.8%) had no formal education. Regarding occupation, most of them were housewives 90 (50%) followed by merchants 30 (16.7%). The majority of the study participants were married, 151 (83.9%). The study participants’ ages ranged from 18 to 45 years old with the median age of 27.0 ± 8.0, 28.0 ± 10.0, and 28.0 ± 9.0 years, for normotensive control, preeclampsia, and eclampsia pregnant women, respectively (Table 1).

**Table 1:** Socio-demographic characteristics of study participants attending at the UoGCSH and FHCSH ANC units and maternity wards, Northwest Ethiopia.

Variables	Categories	Study participants(n = 180)			Total
		Normotensive (n = 60)	Preeclampsia (n = 60)	Eclampsia (n = 60)	
Median Age	–	27.0 ± 8	28.0 ± 10	28.0 ± 9	–
Residence	Urban	48 (80%)	43 (71.7%)	30 (50%)	121 (67.2%)
	Rural	12 (20%)	17 (28.3%)	30 (50%)	59 (32.8%)
Marital status	Single	10 (16.7%)	5 (8.3%)	5 (8.3%)	20 (11.1%)
	Married	47 (78.3%)	51 (85.0%)	53 (88.3%)	151 (83.9%)
	Divorced	3 (5%)	4 (1.7%)	2 (3.3%)	9 (5.0%)
Occupation	House-wife	29 (48.3%)	27 (45%)	34 (56.7%)	90 (50%)
	Student	7 (11.7%)	5 (8.3%)	5 (8.3%)	17 (9.4%)
	Merchant	7 (11.7%)	11 (18.3%)	12 (20%)	30 (16.7%)
	Governmental employee	12 (20%)	9 (15%)	7 (11.7%)	28 (15.6%)
	Unemployed	5 (8.3%)	8 (13.3%)	2 (3.3%)	15 (8.3%)
Educational status	No formal education	7 (11.7%)	12 (20%)	13 (21.7%)	32 (17.8%)
	Primary	23 (38.3%)	12 (20%)	16 (26.7%)	51 (28.3%)
	Secondary	8 (13.3%)	15 (25%)	20 (33.3%)	43 (23.9%)
	College and above	22 (36.7%)	21 (35%)	11 (18.3%)	54 (30%)

### Clinical and anthropometric characteristics of study participants

Among the study participants, 92 (51.1%), and 171 (95%), were primigravida, and nulliparous, respectively. In this study, no statistically significant differences were observed between the case and control groups in terms of gravidity, parity, and MUAC. However, there was a significant difference found between the groups in gestational age, SBP, DBP, and BMI (P < 0.001) (Table 2).

**Table 2:** Clinical and anthropometric characteristics of study participants attending at the UoGCSH and FHCSH ANC units and maternity wards, Northwest Ethiopia.

Variables	Categories	Study participants (N = 180)			p-value
		Normotensive (n = 60)	Preeclampsia (n = 60)	Eclampsia (n = 60)	
Gravidity	Primigravida	28 (46.7%)	33 (55%)	31 (51.7%)	0.655
	Multigravida	32 (53.3%)	27 (45%)	29 (48.3%)	
Parity	Nulliparous	28 (46.7%)	31 (51.7%)	29 (48.3%)	0.113
	Primiparous	14 (23.3%)	6 (10%)	5 (8.3%)	
	Multiparous	18 (30%)	23 (38.3%)	26 (43.3%)	
GA (weeks)	–	34.0 ± 8	37.0 ± 5	37.5 ± 5	<0.001
SBP (mmHg)	–	100.0 ± 14	150.0 ± 16	160.0 ± 20	<0.001
DBP (mmHg)	–	65.0 ± 10	100.0 ± 15	110 ± 5	<0.001
MUAC (cm)	–	23.99 ± 3.1	24.50 ± 3.0	24.0 ± 2.8	0.144
BMI (Kg/m^2^)	–	22.27 ± 2.91	23.46 ± 3.49	25.68 ± 2.64	<0.001
Proteinuria	–	–	1.0 ± 2.0	2.0 ± 1	<0.001

GA: gestational age, SBP: (systolic blood pressure), and DBP: diastolic blood pressure, MUAC: Mid-upper arm circumference, BMI: Body mass index. p-value: chi-square for categorical data and Kruskal wallis test for continuous variables.

### Nutritional characteristics of study participants

Around 147 (81.7%) of the study participants had the feeding habit of meat. One hundred fifty-five (86.1%) and 148 (82.2%) had the habit of feeding vegetables and fruits, respectively. Regarding meat feeding frequency, the majority of study participants, 128 (86.5%), ate less than every two weeks (Table 3).

**Table 3:** Nutritional characteristics of study participants attending the UoGCSH and FHCSH ANC units and maternity wards, Northwest Ethiopia.

Variables	Categories	Study participants (n = 180)		
		Normotensive (n = 60)	Preeclampsia (n = 60)	Eclampsia (n = 60)
Meat feeding habit	Yes	54 (90.0%)	46 (76.7%)	47 (78.3%)
	No	6 (10.0%)	14 (23.3%)	13 (21.7%)
Meat feeding frequency (n = 148)	< 2 weeks	46 (31.1%)	40 (27.0%)	42 (28.4%)
	> 2 weeks	8 (5.4%)	7 (4.7%)	5 (3.4%)
Vegetable feeding habit	Yes	52 (86.7%)	54 (90.0%)	49 (81.7%)
	No	8 (13.3%)	6 (10.0%)	11 (18.3%)
Vegetable feeding frequency (n = 155)	< 2 weeks	49 (31.6%)	53 (43.2%)	49 (31.6%)
	> 2 weeks	3 (1.9%)	1 (0.6%)	0 (0.0%)
Fruit feeding habit	Yes	53 (88.3%)	52 (86.7%)	43 (71.7%)
	No	7 (21.9%)	8 (13.3%)	17 (28.3%)
Fruit feeding frequency (n = 148)	< 2 weeks	44 (29.7%)	46 (31.1%)	39 (26.4%)
	> 2 weeks	9 (6.1%)	6 (4.1%)	4 (2.7%)

### The comparison of coagulation profiless among pregnant women with preeclampsia, eclampsia, and normotensive groups

PT and aPTT data exhibited non-normal distributions. For the preeclampsia, eclampsia patients, and healthy normotensive pregnant women, the median and IQR of the PT values were 18.27 ± 11.08 sec, 19.74 ± 13.61 sec, and 14.45 ± 4.11 sec, respectively. Similarly, the median and IQR of the aPTT values for the preeclampsia, eclampsia patients, and healthy normotensive pregnant women were 35.75 ± 13.5 sec, 35.77 ± 12.22 sec, and 32.56 ± 8.68 sec, respectively. In a Kruskal-Wallis pairwise comparison test, the median and IQR of PT and aPTT in the Preeclampsia and Eclampsia cases were significantly prolonged than the healthy normotensive pregnant women group (p < 0.05). On the other hand, the difference in PT and aPTT between the preeclampsia and eclampsia groups were not statistically significant (p = 0.714) and (p = 0.714) respectively (Table 4).

**Table 4:** Mean comparison of coagulation and platelet parameters of study participants at the UoGCSH and FHCSH ANC units and maternity wards, Northwest Ethiopia.

Parameters	Normotensive (n = 60)	Preeclampsia (n = 60)	Eclampsia (n = 60)	p-values	^X^p values	^Y^p-values	^Z^p values
PLT (10^9^/L)^a^	228.65 ± 38.70	150.10 ± 46.61	133.50 ± 53.89	<0.001*	<0.001*	<0.001*	0.130
MPV (fl)^a^	10.27 ± 1.18	11.17 ± 1.23	11.62 ± 1.37	<0.001*	<0.001*	<0.001*	0.132
PDW (fl)^a^	15.98 ± 0.71	16.51 ± 0.44	16.71 ± 0.60	<0.001*	<0.001*	<0.001*	0.162
PT (sec)^b^	14.45 ± 4.11	18.27 ± 11.08	19.74 ± 13.61	<0.001*	<0.001*	<0.001*	0.714
aPTT(sec)^b^	32.56 ± 8.68	35.75 ± 13.5	35.77 ± 12.22	0.002*	<0.001*	0.006*	0.504

^a^: Data were expressed as mean ± SD,

^b^: Data were expressed as median (IQR). Analysis was performed using a one-way ANOVA followed by a Tuckey post hoc test and a Kruskal-Wallis H test for normally and non-normally distributed data for comparison groups, respectively.

^X^p-value comparison of Normotensive control and Eclampsia case in a pairwise comparison test;

^Y^p-value-comparison of Preeclampsia case to Normotensive control in a pairwise comparison test;

^Z^p-value-comparison of Preeclampsia to Eclampsia case in a pairwise comparison test. *statistically significant mean or median difference at p < 0.05 levels.

### The comparison of Platelet parameters among pregnant women with preeclampsia, eclampsia, and normotensive groups

The data for platelet count, MPV, and PDW followed normal distributions. The mean PLT count for the preeclampsia, eclampsia patients, and healthy normotensive pregnant women were 150.10 ± 46.61 × 10^9^/L, 133.50 ± 53.89 × 10^9^/L, and 228.65 ± 38.70 × 10^9^/L, respectively. Similarly, the mean MPV for the preeclampsia, eclampsia patients, and healthy normotensive pregnant women were 11.17 ± 1.23 fl, 11.62 ± 1.37 fl, and 10.27 ± 1.18 fl, respectively. Further, the mean PDW for the preeclampsia, eclampsia patients, and healthy normotensive pregnant women were 16.51 ± 0.44 fl, 16.71 ± 0.60 fl, and 15.98 ± 0.71 fl, respectively. In a Tuckey post hoc pairwise comparison test, there was a statistically significantly low mean platelet count in the Preeclampsia and Eclampsia cases as compared to the control group (p < 0.001). However, there was no significant mean platelet count difference between the preeclampsia and Eclampsia groups (p = 0.130), while the mean ± SD of MPV and PDW in the Preeclampsia and eclampsia cases were significantly higher than the control group (p < 0.001). In contrast, there was no significant difference in MPV and PDW between the preeclampsia and Eclampsia cases (p = 0.132) and (p = 0.162), respectively (Table 4).

### Abnormal coagulation parameters among pregnant women with preeclampsia and eclampsia groups

In preeclamptic patients, 50 (83.3%), and 14 (23.4%) of the study participants showed abnormalities in PT, and aPTT respectively. The most common abnormality was prolonged PT, affecting 36 (60.0%) of study participants, followed by shorter PT, observed in 14 (23.3%). Similarly, among eclamptic patients, 48 (80.0%), and 10 (16.7%) of study participants showed abnormalities in PT, and aPTT respectively. The predominant abnormalities were prolonged PT, affecting 34 (56.7%) of study participants, followed by shorter PT, observed in 14 (23.3%) (Table 5).

**Table 5:** Abnormal coagulation parameters of preeclampsia and eclampsia patients at the UoGCSH and FHCSH ANC units and maternity wards, Northwest Ethiopia.

Study participants	Coagulation abnormalities					
	PT			aPTT		
Shorter PT (%)	Prolonged PT (%)	Total (%)	Shorter aPTT (%)	Prolonged aPTT (%)	Total (%)
PE	14 (23.3%)	36 (60.0%)	50 (83.3%)	7 (11.7%)	7 (11.7%)	14 (23.4%)
EC	14 (23.3%)	34 (56.7%)	48 (80.0%)	–	10 (16.7%)	10 (16.7%)

PT: Prothrombin Time, aPTT: activated Partial Thromboplastin Time, PE: Preeclampsia, and EC: Eclampsia.

### Abnormal platelet parameters among pregnant women with preeclampsia and eclampsia groups

The magnitude of thrombocytopenia, high MPV, and high PDW in preeclampsia, and eclampsia were 4 (6.7%), 38 (63.3%), 5 (8.3%), and 19 (31.7%), 39 (65.0%), 12 (20.0%) respectively (Table 6).

**Table 6:** Abnormal platelet parameters of preeclampsia and eclampsia patients at the UoGCSH and FHCSH ANC units and maternity wards, Northwest Ethiopia.

Study participants	Platelet parameter abnormalities					
	PLT		MPV		PDW	
Low (%)	High (%)	Low (%)	High (%)	Low (%)	High (%)
PE	4 (6.7%)	–	–	38 (63.3%)	–	5 (8.3%)
EC	19 (31.7%)	–	–	39 (65.0%)	–	12 (20.0%)

PLT: platelet, MPV: Mean Platelet Volume, PDW: Platelet Distribution Width, NT: PE: Preeclampsia and EC: Eclampsia.

### Correlation of mean arterial pressure with coagulation and platelet parameters among pregnant women with preeclampsia and eclampsia groups

Assessment of the association of coagulation profiles and platelet parameters with the severity of disease and their correlation with the MAP was computed in preeclampsia and eclampsia groups, for those parameters that showed significant differences between PIH groups. Mean arterial pressure showed statistically significant negative correlations with PLT (rho = −0.355) in preeclamptic patients. In contrast, MPV, PDW, PT, and aPTT did not show a significant correlation. Similarly, MAP showed statistically significant negative correlations with PLT (rho = −0.685) in the Eclampsia group. Furthermore, MAP showed statistically significant positive correlations with MPV (rho = 0.329), PDW (rho = 0.399), PT (rho = 0.282), and aPTT (rho = 0.176) in the eclampsia group. (Table 7).

**Table 7:** Spearman’s rank-order correlation of PLT, MPV, PDW, PT, and aPTT with MAP in PIH patients at the UoG-CSH and FHCSH ANC units and maternity wards, Northwest Ethiopia.

Correlation coefficient	Parameters with MAP in Preeclampsia				
	PLT	MPV	PDW	PT	aPTT
Rho	−0.355	−0.053	−0.142	0.007	−0.062
P-value	0.005	0.689	0.278	0.960	0.638
Correlation coefficient	**Parameters with MAP in Eclampsia**				
	PLT	MPV	PDW	PT	aPTT
Rho	−0.685	0.329	0.399	0.282	0.176
P-value	<0.001	<0.001	<0.001	<0.001	0.018

aPTT: Activated partial thromboplastin time, PT: Prothrombin time, PLT: Platelet count, MPV: Mean platelet volume, PDW: Platelet distribution width, rho: Spearman’s rank-order correlation, MAP: Mean arterial pressure. * statistically significant at p-value.

## Discussion

Pregnancy-induced hypertension cases develop various hematological changes, with thrombocytopenia being the most common hematological abnormality [33]. Low platelet count is a common hematological manifestation due to hemodilution, increased platelet consumption, and increased platelet aggregation by increased levels of thromboxane A2 [34]. The imbalance in procoagulant and anticoagulant processes may alter blood platelet parameters and coagulation profiles (PLT count, MPV, PDW, PT, aPTT) in preeclampsia and eclampsia patients [35].

In this study, the median and IQR of PT were prolonged in preeclamptic (18.27 ± 11.08 sec) and eclamptic (19.74 ± 13.61 sec) patients when compared to a normotensive pregnant group (14.45 ± 4.11 sec), and it was a statistically significant difference (P < 0.001). However, prolongation in PT in preeclampsia and eclampsia groups was not a statistically significant difference (P = 0.714). The current study’s findings align with those studies conducted in India [36], and Sudan [37], which reported significant prolonged in PIH patients as compared to normotensive health pregnant women controls. The mechanisms of PT prolongation are related to increased levels of fibrin degradation products, and reduced synthesis of coagulation factors [38]. Contrary to this study, studies conducted in Pakistan [18], and Ghana [39], showed that the PT value between preeclampsia patients and the normotensive pregnant group was not statistically significantly different. The reason for variation could be nutritional or lifestyle factors, study design, genetic variations (between the Pakistan and Ethiopian populations differences), analyzer machines used to measure coagulation profiles, and laboratory methodological variations [18,39].

The current study also showed significantly prolonged aPTT in the Preeclampsia group (35.75 ± 13.5 sec), and the Eclampsia group (35.77 ± 12.22 sec) than the normotensive pregnant group (32.56 ± 8.68 sec). Kruskal Wallis Test revealed that the difference in aPTT among study groups was statistically significant (p = 0.002). However, the difference in aPTT between the Preeclampsia and Eclampsia groups was not statistically significant (p = 0.714).

This findings were consistent with studies done in India [36], and Sudan [37], all of those studies reported significant prolongation of aPTT in PIH patients than control groups. Thus, the abnormalities of coagulation parameters in hypertensive disorders of pregnancy indicate intravascular coagulation [35]. On the other hand, the studies conducted in Pakistan [18], and Ghana [39], indicated that aPTT values among preeclampsia and normotensive pregnant groups were not statistically significant. The reason for variation could be due to population demographics, study design, and sample size differences. For instance, the total sample size of a study done in Pakistan was 84 study participants, significantly smaller than our study’s sample size of 180 study participants.

In this finding, there was statistically significant reduced mean platelet counts in preeclampsia (150.10 ± 46.61 × 10^9^/L), and eclampsia (133.50 ± 53.89 × 10^9^/L) patients, as compared to normotensive healthy pregnant women (228.65 ± 38.70 × 10^9^/L) (P < 0.001). Platelet count was reduced in eclamptic patients compared to preeclampsia patients, but there was no significant difference in mean platelet count between eclampsia and preeclampsia groups (P = 0.130). This shows that with the onset of preeclampsia, platelet count begins to reduce with eclampsia patients having markedly reduced platelet counts. Thus, platelet counts continue to fall significantly with proceedings from preeclampsia to eclampsia. Indicating a significant decrease in mean platelet counts with the increasing grades of pregnancy-induced hypertension cases.

The finding of the current study is consistent with studies conducted in India [36], Iraq [40], Nigeria [41], and Ethiopia [42–44], all of those studies reported significantly decreased mean platelet count in PIH patients as compared to controls. The cause of platelet decrement is multifactorial and is related to hemodilution, increased platelet consumption, and increased platelet aggregation driven by increased levels of thromboxane A2 [45]. Furthermore, related to endothelial damage in preeclampsia and eclampsia, which causes an increase in platelet aggregation and turnover. They indicated an inverse relationship between the severity of PIH and platelet numbers [43]. Contrary to our findings, studies conducted in Nigeria [46], and Pakistan [18], reported that, no platelet count significant difference between normal health control and PIH pregnant women. The reason for variation could be nutritional or lifestyle factors, study design, study period, and differences in the sample size of study participants.

This study revealed that mean MPV in Preeclampsia (11.17 ± 1.23 fL) and Eclampsia (11.62 ± 1.37 fL) patients were significantly higher compared to normotensive healthy pregnant women (10.27 ± 1.18 fL) (P < 0.001). Despite the MPV being higher in eclampsia patients when compared to preeclampsia patients, there was no statistically significant difference (p = 0.132). This shows that there is a significant increase in MPV with the onset of PIH and consequently with preeclamptic and eclamptic patients. Studies done in India [36], Iraq [40], Nigeria [41], and Ethiopia [42–44], found that the mean platelet volume was increased in preeclampsia and eclampsia as compared to normotensive healthy controls. The increment in the MPV value in preeclampsia and eclampsia patients might be due to the presence of large platelets in the peripheral circulation. In patients with preeclampsia and eclampsia, the bone marrow is obligated to produce and release young platelets with large sizes to compensate for the accelerated consumption and destruction of platelets due to aggregation within the damaged endothelium [18,43].

Our results differed from the pairwise comparison of normal and PIH pregnant women studies done in Nigeria [46], and Pakistan [18]. The difference may probably be due to the different sample sizes included in their studies, different equipment, and methods of the automatic blood count analyzers. Additionally, it could be due to lifestyle factors, study design, and sample size. This study showed an increase in PDW in preeclampsia (16.51 ± 0.44 fL) and eclampsia (16.71 ± 0.60 fL) patients when compared to normotensive healthy pregnant women (15.98 ± 0.71 fl) with a statistically significant difference (p-value). Even though PDW was higher in eclamptic patients when compared to preeclampsia patients, there was no statistically significant difference in mean PDW between eclampsia and preeclampsia groups (P = 0.162). The increased PDW in PIH patients could be due to platelet activation as a result of endothelial damage, which may cause a change in shape from discoid to spherical to obtain a larger surface, resulting in an increased PDW in PIH patients [18].

Studies conducted in India [36], Iraq [40], Nigeria [41,46], and Ethiopia [42–44], found that the PDW was increased in preeclampsia and eclampsia as compared to a normotensive pregnant women group. Furthermore, they indicated that there was an association between platelet indices and the severity of preeclampsia. This finding disagreed with studies conducted in Pakistan [18]. The possible disagreement in PDW values might be due to the differences in equipment and method of the automated hematologic analyzer, study design, and genetic variations [36].

### Strengths and limitations of the study

The multicenter design of this study and the inclusion of age and gestational age matched case-control comparators will strengthen the current study, which gives a clue for coagulopathy among preeclampsia and eclampsia in the region. However, it is important to note that there are certain limitations in this study, such as the exclusion of specific coagulation and platelet parameters, such as fibrinogen, D-dimer, TT, plateletcrit, and PLT functional assays. These parameters play a crucial role in assessing coagulation disorders and their omission may limit a comprehensive understanding of the association between PIH and coagulopathy and platelet disorders.

## Conclusion and recommendations

Preeclampsia and eclampsia patients exhibit significantly lower platelet count, whereas, there were higher mean MPV and PDW values compared to normotensive controls. Similarly, significant differences were observed in median PT, and aPTT values between preeclampsia, eclampsia patients, and normotensive pregnant women. Therefore, a routine diagnostic test of coagulation profiles and platelet parameters for preeclampsia and eclampsia patients could be applicable. In addition, we encourage a large-scale and longitudinal studies to further elucidate the distribution of coagulation and platelet parameters among hypertensive pregnant womens.

## Supporting information

S1 Checklist STROBE checklist for observational study. (DOCX). (DOCX)

S1 Table Variable assessment tool. (PDF) (PDF)

## Abbreviations

ANC: Antenatal Care
aPTT: activated Partial Thromboplastin Time
ANOVA: Analysis of Variance
BMI: Body Mass Index
CBC: Complete Blood Count
DBP: Diastolic Blood Pressure
EDTA: Ethylene Diamine Tetraacetic Acid
FHCSH: Felege-Hiwot Comprehensive Specialized Hospital
HBsAg: INR: International Normalization Ratio
MPV: Mean Platelet Volume
PDW: Platelet Distribution Width
PIH: Pregnancy Induced Hypertension
PPP: Platelet Poor Plasma
PT: Porthrombin Time
SBP: Systolic Blood Pressure
UoGCSH: University of Gondar Comprehensive Specialized Hospital
WHO: World Health Organization.
