# Supplementary material for: Coagulation profiles and platelet parameters among preeclampsia, eclampsia, and normotensive pregnant women attending Comprehensive Specialized Hospital maternity wards, Northwest Ethiopia
Source: PLoS One. 2025 Jul 21;20(7):e0328578. doi: 10.1371/journal.pone.0328578 (PMC12279111; doi:10.1371/journal.pone.0328578)
Supplement: S1 Checklist — (DOCX) [file pone.0328578.s001.docx]

**S1 Checklist: STROBE checklist for observational study**

|  | Item No. | Recommendation | Page No | Relevant text from the manuscript |
| --- | --- | --- | --- | --- |
| **Title and abstract** | 1 | (a) Indicate the study’s design with a commonly used term in the title or the abstract | 1 | Comparative cross-sectional study |
|  |  | (b) Provide in the abstract an informative and balanced summary of what was done and what was found | 1-2 |  |
| Introduction | | | |  |
| Background/rationale | 2 | Explain the scientific background and rationale for the investigation being reported | 2-5 |  |
| Objectives | 3 | State specific objectives, including any pre-specified hypotheses | 5 |  |
| Methods | | | |  |
| Study design | 4 | Present key elements of study design early in the paper | 6 |  |
| Setting | 5 | Describe the setting, locations, and relevant dates, including periods of recruitment, exposure, follow-up, and data collection | 6 |  |
| Participants | 6 | Cross-sectional study—Give the eligibility criteria, and the sources and methods of selection of participants | 6-7 |  |
| Variables | 7 | Clearly define all outcomes, exposures, predictors, potential confounders, and effect modifiers. Give diagnostic criteria, if applicable |  | NA |
| Data sources/ measurement | 8* | For each variable of interest, give sources of data and details of methods of assessment (measurement). Describe comparability of assessment methods if there is more than one group | 8-10 | A total of 8 mL venous blood was collected by syringe and needle method, 3 ml of blood have been added to K_2_-EDTA 3 mL of blood have been added to 3.2% trisodium citrate tube  2 ml of blood have been added to the SST for HIV, and hepatitis B and C virus screening tests. |
| Bias | 9 | Describe any efforts to address potential sources of bias |  | NA |
| Study size | 10 | Explain how the study size was arrived at | 8 | Sample size was calculated using the rules of thumb that have been recommended by van Voorhis and Morgan, 30 participants per group are required to detect real differences, which can lead to about 80% power in a comparative study. |

| Quantitative variables | 11 | Explain how quantitative variables were handled in the analyses. If applicable, describe which groupings were chosen and why | 8 |  |
| --- | --- | --- | --- | --- |
| Statistical methods | 12 | (a) Describe all statistical methods, including those used to control for confounding | 8 |  |
|  |  | (b) Describe any methods used to examine subgroups and interactions |  | NA |
|  |  | (c) Explain how missing data were addressed | 8 |  |
|  |  | (d) Cross-sectional study—If applicable, describe analytical methods taking account of sampling strategy |  | NA. |
|  |  | (e) Describe any sensitivity analyses |  | NA |
| Results | | | | |
| Participants | 13* | (a) Report numbers of individuals at each stage of study—eg numbers potentially eligible, examined for eligibility, confirmed eligible, included in the study, completing follow-up, and analysed |  | NA |
|  |  | (b) Give reasons for non-participation at each stage |  | NA |
|  |  | (c) Consider use of a flow diagram |  | NA |
| Descriptive data | 14* | (a) Give characteristics of study participants (eg demographic, clinical, social) and information on exposures and potential confounders | 13 |  |
|  |  | (b) Indicate number of participants with missing data for each variable of interest |  | NA |
| Outcome data | 15* | Cross-sectional study—Report numbers of outcome events or summary measures | 13-17 |  |
| Main results | 16 | (a) Give unadjusted estimates and, if applicable, confounder-adjusted estimates and their precision (eg, 95% confidence interval). Make clear which confounders were adjusted for and why they were included | 13-17 |  |
|  |  | (b) Report category boundaries when continuous variables were categorized |  | NA |
|  |  | (c) If relevant, consider translating estimates of relative risk into absolute risk for a meaningful time period |  | NA |

Continued on next page

| Other analyses | 17 | Report other analyses done—eg analyses of subgroups and interactions, and sensitivity analyses |  | NA |
| --- | --- | --- | --- | --- |
| Discussion | | | | |
| Key results | 18 | Summarise key results with reference to study objectives | 17-21 | The findings showed that the Preeclampsia and eclampsia patients exhibit significantly lower platelet count, whereas, there were higher mean MPV and PDW values compared to normotensive healthy controls. Similarly, significant differences were observed in median PT, and aPTT values between preeclampsia, eclampsia patients, and normotensive pregnant women |
| Limitations | 19 | Discuss limitations of the study, taking into account sources of potential bias or imprecision. Discuss both direction and magnitude of any potential bias | 22 | Exclusion of specific coagulation and platelet parameters, such as fibrinogen, D-dimer, TT, plateletcrit, and PLT functional assays |
| Interpretation | 20 | Give a cautious overall interpretation of results considering objectives, limitations, multiplicity of analyses, results from similar studies, and other relevant evidence | 22 |  |
| Generalisability | 21 | Discuss the generalisability (external validity) of the study results | 22 |  |
| Other information | |  | | |
| Funding | 22 | Give the source of funding and the role of the funders for the present study and, if applicable, for the original study on which the present article is based | 23 |  |
