# Supplementary material for: Coagulation profiles and platelet parameters among preeclampsia, eclampsia, and normotensive pregnant women attending Comprehensive Specialized Hospital maternity wards, Northwest Ethiopia
Source: PLoS One. 2025 Jul 21;20(7):e0328578. doi: 10.1371/journal.pone.0328578 (PMC12279111; doi:10.1371/journal.pone.0328578)
Supplement: S1 Table — (PDF) [file pone.0328578.s002.pdf]

S1 Table: Variable assessment questionnaire

Socio-demographic characteristics		
S/N	Variables	Response
101	Age (in years)	_________
102	Residence	1. Urban<br>2. Rural
103	Marital status	1. Single<br>2. Married<br>3. Divorced<br>4. Widowed
104	Educational status	1. No formal education<br>2. Primary school<br>3. Secondary school<br>4. College and above
105	Occupational status	1. Housewife<br>2. Student<br>3. Merchant<br>4. Government employee<br>5. Others_______________

Clinical and anthropometric characteristics		
S/n	Variables	Results
201	Gestational age (in weeks)	________
202	Frequency of pregnancy	1. Once (primigravida)<br>2. More than once (multigravidas)
203	Parity	1. Nulliparous<br>2. Primiparous<br>3. Multiparous
204	Height (in meters)	_________
205	Weight (in kilograms)	_________
206	Body mass index (Kg/m^2)	_________
207	MUAC (in centimetres)	_________
208	Blood pressure (mmHg)<br>Systolic blood pressure<br>Diastolic blood pressure	mmHg<br>_________<br>_________
209	Degree of Proteinuria (0, +1, +2…)	_________

**Nutritional assessment parameters**		
S/N	Questions	Response
301	Meat eating habit	1. Yes ☐<br>2. No ☐
302	If "Yes", frequency of meat feeding	1. Daily ☐ 2. Every two days ☐<br>3. Every two weeks ☐ 4. Once a month ☐
303	Vegetables eating habits	1. Yes ☐<br>2. No ☐
304	If "Yes", frequency of vegetable feeding	1. Daily ☐ 2. Every two days ☐<br>3. Every two weeks ☐ 4. Once a month ☐
305	Fruit-eating habit	1. Yes ☐<br>2. No ☐
306	If "Yes", frequency of Fruit feeding	1. Daily ☐ 2. Every two days ☐<br>3. Every two weeks ☐ 4. Once a month ☐
